# Analgesic Efficacy of Phytotherapeutic Agents in Dental Pain Management: A Systematic Review

**DOI:** 10.1155/ijod/5614623

**Published:** 2025-11-20

**Authors:** Manchala Sesha Reddy, Srinivas Sulugodu Ramachandra, Shishir Ram Shetty, Shakeel S. Khazi, Mohammed Shoaib ur Rahman Tippu, L. Ashwin Narayanan, Raghavendra Manjunath Shetty, Sabrin A. Azim, Vannala Venkata Ramana, Sura Ali Ahmed Fuoad Al-Bayati, Ali Razooki, Nader Nabil Fouad

**Affiliations:** ^1^Preventive Dental Department, College of Dentistry, CUA, Ajman, UAE; ^2^Department of Restorative Dentistry, University of Sharjah College of Dental Medicine, Sharjah, UAE; ^3^Department of Oral Craniofacial Health Sciences, University of Sharjah College of Dental Medicine, Sharjah, UAE; ^4^Department of Restorative Dentistry, College of Dentistry, Gulf Medical University, Ajman, UAE; ^5^Department of Surgical Sciences, College of Dentistry, Gulf Medical University, Ajman, UAE; ^6^Department of Preventive Dentistry, Ajman University of Science and Technology College of Dentistry, Ajman, UAE; ^7^Department of Orthodontics, Panineeya Institute of Dental Sciences, Hyderabad, Telangana State, India; ^8^Restorative and Prosthodontic Dentistry, College of Dentistry, CUA, Ajman, UAE; ^9^Oral and Maxillofacial Diagnosis and Surgery, College of Dentistry, CUA, Ajman, UAE

**Keywords:** analgesic, herbal medicine, phytotherapy, plant extract, toothache, VAS

## Abstract

**Background:**

Toothache represents one of the most common reasons patients seek emergency dental care. While conventional analgesics remain the standard of care, phytotherapeutic agents offer potential alternatives with possibly fewer side effects. This systematic review evaluates the efficacy, safety, and clinical applications of plant-derived analgesic compounds in dentistry.

**Methods:**

A comprehensive search was conducted across electronic databases, including PubMed, Cochrane Library, Scopus, and Web of Science, for studies published between 15^th^ January 2015–25^th^ March 2025. Keywords included combinations of “phytotherapy,” “herbal medicine,” “plant extract,” “toothache,” “analgesic,” “visual analog scale (VAS),” and “dentistry.” Randomized controlled trials (RCTs), clinical trials, and systematic reviews were included. The quality of evidence was assessed using the GRADE approach.

**Results:**

A total of 21 studies met the inclusion criteria. The phytotherapeutic agents demonstrating the strongest analgesic properties in dental applications include clove oil (eugenol), turmeric (curcumin), capsaicin from chili peppers, ginger powder, capsaicin, jidabokuippo, cannabis, propolis, *zingiber officinale*, green tea, and *punica granatum*. Mechanisms of action range from inhibition of prostaglandin synthesis to modulation of inflammatory pathways and direct effects on nociceptors. Clinical applications included management of pulpitis, postextraction pain, temporomandibular disorders, and oral mucositis.

**Conclusion:**

Evidence supports the analgesic efficacy of several phytotherapeutic agents in toothache management, particularly eugenol, curcumin, and capsaicin. These offer promising alternatives or adjuncts to conventional analgesics, though standardization of preparations and larger RCTs are needed to establish optimal dosing regimens and long-term safety profiles.

## 1. Introduction

Plants have long been essential to human survival, serving not only as sources of food, clothing, and shelter, but also as medicines. Healing traditions such as Ayurveda, Unani, and traditional Chinese Medicine rely heavily on medicinal plants, and their use remains especially prominent in Asia and Africa. In recent years, however, plant-based remedies have also gained attention in developed countries largely because they are affordable and accessible. According to the World Health Organization (WHO), nearly 80% of the global population continues to depend on traditional medicine for basic healthcare [1–4].

The word *phytotherapy* is derived from the Greek *phyton* (plant) and *therapeia* (treatment) and refers to the prevention and treatment of diseases through plant-based remedies. Records suggest that the practice dates back as early as 4000 bc across different cultures [5, 6]. Beyond managing illness, phytotherapy relieves suffering and promotes recovery. In addition, many plant extracts exhibit antibacterial, anti-inflammatory, antihemorrhagic, and anesthetic effects, making them promising as complementary or alternative therapeutic options [7]. With antimicrobial resistance emerging as a major global concern, natural approaches such as phytotherapy are receiving renewed attention [8].

Plants have also provided the foundation for modern drug discovery; roughly around one-quarter of modern medicines are derived, directly or indirectly, from plant compounds. Phytotherapeutic products are currently available as tablets, solutions, gels, and ointments, and are increasingly prescribed by healthcare professionals [9, 10]. In dentistry, interest in herbal medicines has grown rapidly, with research focusing on their potential to relieve pain, reduce inflammation, control infections, stimulate healing, and support tissue regeneration. Compared to synthetic drugs, plant-based options are often less toxic, more biocompatible, and more cost-effective [11].

**Figure 1:**
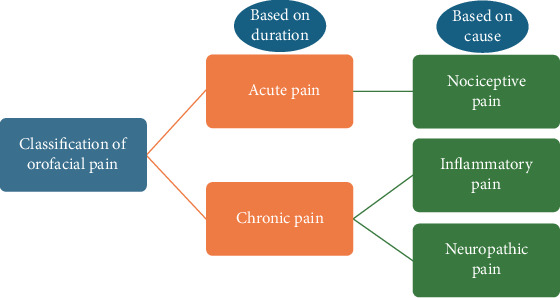
A schematic showing the broad classification of pain, with pain being classified as acute and chronic pain based on the duration of pain, whereas, based on the cause, pain can be classified as nociceptive, inflammatory, and neuropathic pain.

Despite global preventive efforts, oral diseases remain one of the most common health burdens, affecting an estimated 3.5 billion people worldwide [12–14]. The most prevalent conditions include dental caries, periodontitis, oral cancers, oral manifestations of systemic diseases, trauma, and noma in children. Inflammation is a hallmark of these conditions and a major contributor to the pain and discomfort they cause [14, 15]. Pain and inflammation are closely intertwined: pain signals tissue damage and inflammation, representing the body's defense. Dental pain can range from sharp and sudden to persistent and dull, and is often associated with caries, pulp or nerve involvement, or periodontal disease. It also commonly occurs after dental treatments such as extractions or endodontic procedures [16–18] Figure 1.

Conventional pain medications, including nonsteroidal anti-inflammatory drugs (NSAIDs), work by blocking cyclooxygenase (COX) enzymes that produce prostaglandins, responsible for pain and inflammation. While effective, these drugs have notable drawbacks: NSAIDs can cause gastric ulcers, COX-2 inhibitors may increase cardiovascular risks, and opioids carry the risk of dependence. These limits have heightened interest in plant-based alternatives, which may offer good analgesic and anti-inflammatory benefits with reduced side effects [19, 20].

This review synthesizes current scientific evidence on the role of herbal medicines in dentistry, with a particular emphasis on phytochemicals and plant extracts that show promise in managing dental pain.

## 2. Materials and Methods

Though the research protocol for the systematic review was not registered in any of the registries (PROSPERO), the systematic review involved preset inclusion and exclusion criteria and ensured that all the steps involved in a systematic review were followed to ensure transparency and to avoid author bias.

### 2.1. Search Strategy

A comprehensive search was conducted across PubMed, Cochrane Library, Scopus, and Web of Science, covering studies published between 15 January 2015 and 25 March 2025. The last search was conducted on 25 March 2025. Keywords included combinations of “phytotherapy,” “herbal medicine,” “plant extract,” “analgesic,” and “dentistry-related pain outcomes (Supporting Information 1).”

All Boolean operators (e.g., AND, OR, NOT) used in the search were written in uppercase to follow database search conventions. The databases searched were PubMed, Cochrane Library, Scopus, and Web of Science, with consistent reporting across the manuscript (Supporting Information 2).

A comprehensive search was conducted using keywords like “phytotherapy,” “herbal medicine,” “plant extract,” “botanical,” “toothache,” “analgesic,” “analgesia,” “pulpitis,” “dentistry,” “oral pain,” “postextraction,” “VAS,” and “temporomandibular disorder.” These terms were chosen based on the relevant literature and Medical Subject Headings (MeSH) terms. The search spanned multiple databases, including PubMed, Cochrane Library, Scopus, and Web of Science, covering English-language publications to ensure the inclusion of recent and clinically relevant evidence reflecting current advances in research and practice, while excluding outdated methodologies.

### 2.2. Inclusion and Exclusion Criteria

#### 2.2.1. Inclusion Criteria

Randomized controlled trials (RCTs), controlled clinical trials, or systematic reviews/meta-analyses published in English evaluating the analgesic effects of phytotherapeutic agents in dental applications undertaken on humans were included. Additionally, the studies needed to have clearly defined outcome measures for pain assessment, including a visual analog scale (VAS), a modified VAS (MVAS), a numeric rating scale (NRS), or a perceived pain rating scale.

#### 2.2.2. Exclusion Criteria

Studies focusing exclusively on antimicrobial properties without pain assessment, studies that included only pediatric patients, focusing on dentinal hypersensitivity, and those using nonvalidated tools for pain assessment were excluded. Additionally, case reports, opinion papers, and narrative reviews were excluded.

### 2.3. Study Selection and Data Extraction

Two independent reviewers (SMR and SSR) screened the titles and abstracts of all identified studies for eligibility according to predetermined criteria (Kappa score 0.80, indicating good agreement). Full-text articles of potentially eligible studies were retrieved and independently assessed (Kappa score 0.80, indicating good agreement). Disagreements were resolved through discussion or consultation with a third reviewer (SRS), if necessary. Data extraction was performed using a standardized form that captured the following information: study characteristics (authors, year, country, study design), participant demographics and sample size, type and formulation of phytotherapeutic agents, intervention and control protocols, pain assessment methods, and timepoints.

### 2.4. Quality Assessment and Risk of Bias Indicators

The methodological quality of included RCTs was assessed using the Cochrane Risk of Bias tool, which evaluated the included RCTs under the following individual domains: random sequence generation, allocation concealment, blinding of participants and personnel, blinding of outcome assessment, incomplete outcome data, selective reporting, and other sources of bias [21]. The overall quality of evidence for each phytotherapeutic agent was evaluated using the Grading of Recommendations Assessment, Development and Evaluation (GRADE) approach, considering risk of bias, inconsistency, indirectness, imprecision, and publication bias. A summary of findings table was created to present the overall certainty of evidence (High, Moderate, Low, and Very Low) for each key outcome, in accordance with GRADE guidelines.

### 2.5. Data Synthesis

Given the anticipated heterogeneity in study designs, interventions, and outcome measures, a narrative synthesis approach was adopted. Where possible, studies were grouped by the type of phytotherapeutic agent and dental condition.

## 3. Results

**Figure 2:**
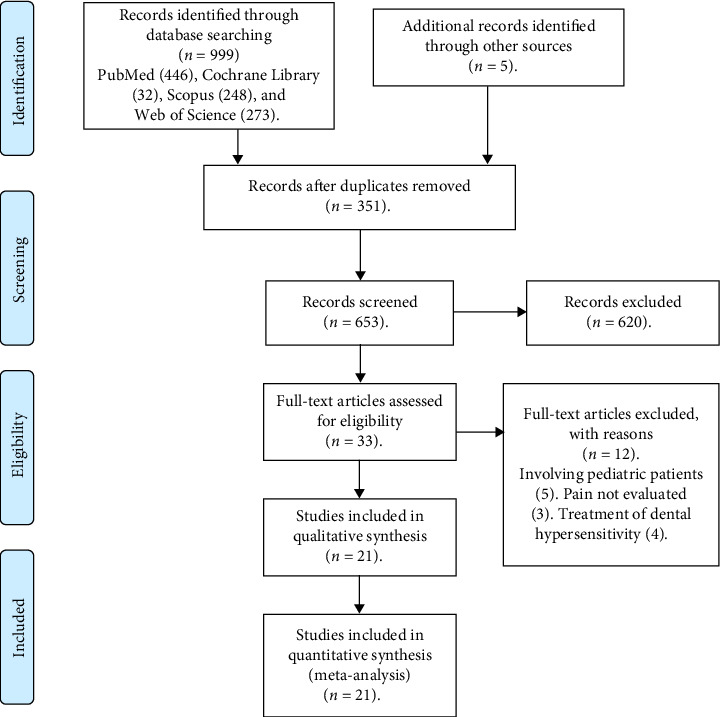
PRISMA flow diagram of study selection for the systematic review on phytotherapeutic agents for pain reduction in dental treatment.

The literature search identified 999 records from electronic databases (PubMed = 446, Cochrane Library = 32, Scopus = 248, Web of Science = 273) and an additional five records through manual searching. After duplication, 653 records remained for title and abstract screening. Of these, 620 records were excluded, leaving 33 full-text articles for eligibility assessment. Following detailed evaluation, 12 articles were excluded with reasons (5 involved pediatric patients, 3 did not evaluate pain outcomes, and 4 investigated dental hypersensitivity). Ultimately, 21 studies met the inclusion criteria and were incorporated into the qualitative synthesis. The study selection process is presented in Figure 2 (PRISMA 2020 flow diagram), which details the number of records identified, screened, excluded (with reasons), and included. The main characteristics of the included studies are summarized in Table 1. This report follows the PRISMA 2020 checklist to ensure transparent and comprehensive documentation of the review process.

Of the 21 included studies, nine were RCTs, one was a single-blind study, seven were double-blind studies, two were split-mouth studies, and one crossover split-mouth RCT. The studies were conducted in 18 countries, with the majority from India (*n* = 4), Iran (*n* = 4), Saudi Arabia (*n* = 2), United States (*n* = 2), Brazil (*n* = 1), China (*n* = 1), Japan (*n* = 1), Indonesia (*n* = 1), Poland (*n* = 2), Spain (*n* = 1), Pakistan (*n* = 1), and France (*n* = 1), The sample sizes in these studies ranged from 10 to 270 participants.

The dental conditions addressed included pain due to periodontal origin (*n* = 6), endodontic origin (*n* = 4), postextraction pain (*n* = 5), pain associated with temporomandibular disorders (*n* = 3), and oral mucositis-associated pain (*n* = 3). Among the studies included, pain assessment methods varied, with the VAS being the most used (*n* = 14), followed by the NRS (*n* = 5), MVAS (*n* = 1), and verbal rating scale (VRS) (*n* = 1). Table 1

### 3.1. Risk of Bias Assessment

**Figure 3:**
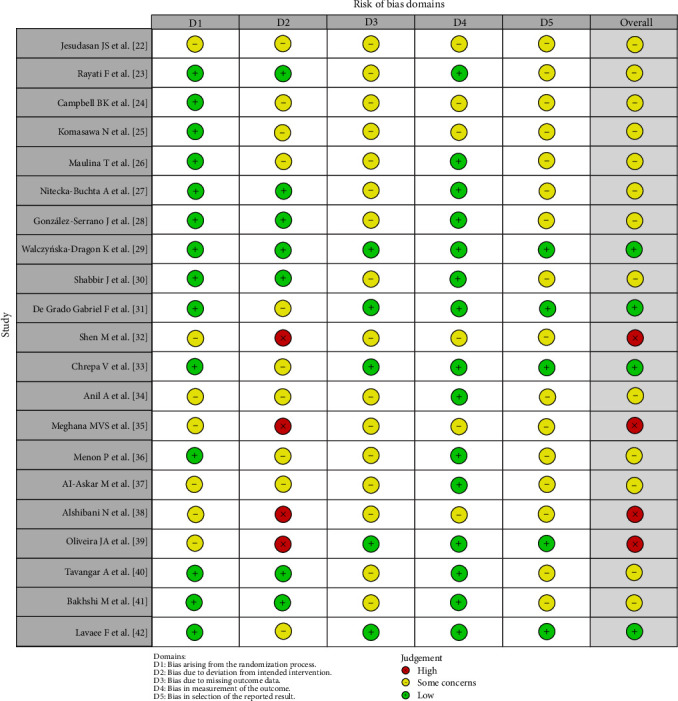
Risk of bias assessment of studies evaluating phytotherapeutic agents for pain management in dental treatment.

**Figure 4:**
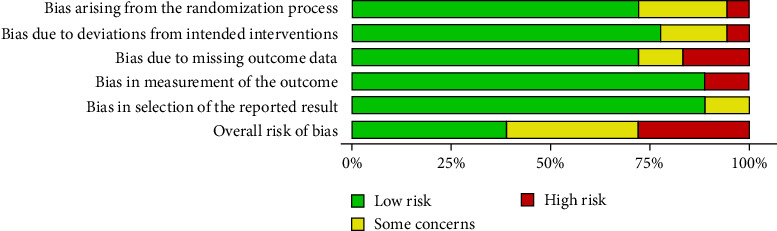
RoB assessment: summary plot.

Based on the risk of bias assessment, only one study was identified as having a low risk of bias. A majority of 16 studies were found to have some concerns, indicating an unclear risk of bias, while four studies demonstrated a high risk of bias. Figures 3 and 4 present the risk of bias assessment for the studies included across the five evaluated domains. Along with the study-level risk of bias assessment (Figures 3 and 4), we used the GRADE approach to rate the certainty of evidence. The summary of findings is provided in Table 2.

### 3.2. Phytotherapeutic Agents With Analgesic Properties in Dentistry

In oral surgery, eugenol reduced postextraction pain compared to chlorhexidine for alveolar osteitis prevention [22, 31, 32]. Curcumin was tested in several studies with variable outcomes; some trials showed equivalence to mefenamic acid, while others reported limited benefit depending on formulation [26, 34, 35, 37, 41]. Cannabis-derived cannabidiol (CBD) demonstrated significant pain reduction in temporomandibular disorders and emergency toothache, with dose-dependent effects [27, 29, 33]. Ginger showed consistent analgesic effects, comparable to ibuprofen, in both postsurgical and periodontal pain management [23, 36].

The description of phytotherapeutic agents has been harmonized to ensure consistent reporting of efficacy, mechanism of action, clinical applications, and safety profiles across all agents discussed.

### 3.3. Clinical Efficacy by Dental Specialty

Upon assessment of the studies based on the dental specialty, oral surgery applications had eight studies investigating phototherapeutic agents for postsurgical pain management, with eugenol, ginger, and curcumin showing promising results. Eugenol demonstrated efficacy in preventing alveolar osteitis, while ginger powder achieved pain control equivalent to conventional NSAIDs [22, 29]. For endodontic applications, four studies focused on postprocedural endodontic pain, with eugenol and propolis showing significant benefits. CBD demonstrated effectiveness for emergency acute toothache, providing an alternative to conventional analgesics [30–33]. For periodontal applications, six studies evaluated postsurgical periodontal pain, with mixed results for curcumin but consistent benefits for ginger. The variation may relate to formulation differences and delivery methods [34–39]. For oral medicine applications, three studies investigated recurrent aphthous stomatitis, with *Punica granatum* showing consistent benefits across multiple formulations [40–42].

## 4. Discussion

In the past two decades, there has been an increasing interest in exploring natural and plant-based derivatives owing to their efficacy and purported lack of side effects. The potential development of antimicrobial resistance and the antimicrobial stewardship policies have minimized the use of antimicrobials for dental diseases, forcing clinicians to look for suitable alternatives, primarily plant-based derivatives. Among the 21 included clinical studies, the role of eugenol and ginger was consistent, with curcumin and CBD showing promising outcomes. This systematic review identified 21 clinical studies evaluating phytotherapeutic agents for dental pain management. The most consistent evidence supports eugenol and ginger, while curcumin and cannabidiol (CBD) showed variable but promising outcomes. Propolis, *Punica granatum*, and green tea extract demonstrated limited evidence.

These mechanisms of action of phytotherapeutic agents act through diverse pathways, including prostaglandin inhibition (eugenol, curcumin, ginger), receptor modulation (CBD, capsaicin), and antioxidant/anti-inflammatory effects (propolis, *Punica granatum*, green tea). A key factor is that these mechanisms are complementary to the role of conventional analgesics, thus having a synergistic effect among patients. These mechanisms complement conventional analgesics. Topical capsaicin or jidabokuippo preparations should be considered for persistent temporomandibular pain and neuropathic orofacial pain conditions that do not respond adequately to standard therapies [24, 25].

Eugenol was consistently effective across oral surgery and endodontic pain, with moderate-certainty evidence [31, 43, 44]. Curcumin showed variable outcomes influenced by formulation and bioavailability, with low-certainty evidence [33, 37, 41, 45]. Ginger is comparable to ibuprofen for postsurgical and periodontal pain, with its demonstration of moderate-certainty evidence [23, 36]. CBD demonstrated dose-dependent pain reduction in TMD and emergency toothache, with low-to-moderate certainty of evidence [27, 29, 33]. Small-sample studies suggest that Propolis and Punica granatum may offer analgesic benefits; however, these findings are preliminary and require confirmation in larger, well-designed trials [28, 30, 40, 42]. In contrast, the evidence for green tea is low to very low in certainty and shows no consistent analgesic effect [39].

Some of these agents have been in use in different forms and formulations historically. Therefore, the clinical translation of these agents, specifically those with positive findings, seems feasible. Clinical applications and implications: clinical translation is feasible for selected agents. The use of eugenol for pain relief in pulpitis and in the prevention of Eugenol is effective for confirms previously established findingsalveolar osteitis prevention and pulpal pain relief [31, 43]. Ginger can substitute NSAIDs in patients with contraindications [23, 36]. Patients with a history of gastrointestinal issues, peptic ulcers, increased bleeding risk, and kidney problems may be better served with the delivery of these plant-based products instead of NSAIDs. Curcumin formulations may offer benefit in periodontal pain when optimized for delivery [37, 41]. Patients with orofacial pain are known to be on several analgesics or a cocktail of analgesics owing to several reasons, including lack of definitive diagnosis and the multifactorial nature of the disease. The potential of CBD to act centrally on the cannabinoid receptors, part of the endocannabinoid system, is known to provide positive results. Since the CBD has no addictive potential due to the lack of tetrahydrocannabinol, this agent may emerge as an alternative analgesic for may emerge as an alternative analgesic for orofacial pain in the future [29, 33]. The use of CBD agents is currently being explored for a variety of purposes, and Propolis and *Punica granatum* show potential as adjuncts for oral mucosal pain but require further evidence [28, 30].

This systematic review has several limitations. First, the review protocol was not registered in a systematic review registry. However, the review followed the PRISMA guidelines to ensure transparency and reproducibility of the findings. Second, the lack of funding for translation and logistical issues forced the authors to include articles published in the English language only, resulting in the exclusion of a few articles that may exist in languages other than English. Third, the lack of article search in gray literature may also have led to the noninclusion of a few articles. Finally, a search period of the past 10 years may have resulted in the exclusion of articles before the period, as interest in plant-based products existed more than two decades ago.

The lack of standardization in the extraction, formulation, and dosage of these agents makes comparison of the data challenging. Studies with a small sample size and heterogeneity in pain measurement also add to the complexity of data comparison. Future studies should be carried out with high methodological rigor and a sufficient sample size, following the established pharmacopeia standards. This will allow the comparison of the products across different research designs, allowing for potential clinical practice guidelines for the use of phytotherapeutic agents for dental pain relief. Regulatory and standardization challenges variability in extraction, formulation, and dosage limit reproducibility. Establishing pharmacopeial standards and ensuring quality control are critical for these steps in future scientific publications may smooth the integration of phytotherapy into evidence-based dental practice. Methodological Limitations are that most studies had small sample sizes (< 60 participants), short follow-up periods, and heterogeneity in pain measurement tools. Publication bias cannot be excluded, and language restrictions may have excluded relevant evidence. Future research should focus on large, multicenter RCTs with standardized formulations, studies evaluating the pharmacokinetics–pharmacodynamics of the phytotherapeutic agent, dose-finding studies, and pharmacokinetic investigations. Comparative trials against conventional analgesics and combination therapies should be prioritized. Inclusion of patient-centered outcomes, cytokine levels from the local sites, long-term safety data, and cost-effectiveness in the research design will enhance the quality of the studies. Studies should also assess long-term safety, cost-effectiveness, and patient-centered outcomes.

## 5. Conclusion

Phytotherapeutic agents, particularly eugenol and ginger, offer clinically relevant analgesic benefits for dental pain management in dentistry. While curcumin, CBD, propolis, and *Punica granatum* hold promise, further standardized, high-quality trials are essential. Integration into clinical practice will require regulatory frameworks and robust evidence to support safety, efficacy, and reproducibility.

## Figures and Tables

**Table 1:** Phytotherapeutic agents with analgesic properties used in dentistry.

S. no.	Authors	Agent	Sample size/study type	Mode of delivery/pain response	Dosage	Dental procedure/treatment	Human studies	Mechanism of action	Limitations
Oral surgery									
1	Jesudasan et al. [22]	Eugenol	270 patients/RCT	Gel/paste/VAS	0.2% chlorhexidine (CHX) gel vs. eugenol-based. Paste vs. controls. No treatment.	Treat alveolar osteitis.	Authors stated that eugenol was the best of the 2 interventions to treat alveolar osteitis.	Nerve block + reduced inflammation.	Short follow-up and single application.
2	Rayati et al. [23]	Ginger powder	67 patients/double blind RCT	Tablet/powder/VAS	Ibuprofen vs. ginger. Powder vs. placebo	Postsurgical pain after 3^rd^ molar extraction.	Researchers reported that ginger powder is as effective as ibuprofen in the management of the postsurgical sequelae.	Inhibiting both COX and LOX pathways.	Not reported.
3	Campbell et al. [24]	Capsaicin	44 patients/RCT	Cream application/VAS	8% capsaicin vs. vehicle cream.	Temporomandibular disorders (TMJ pain).	Authors reported that 8% topical capsaicin therapy is a relatively safe, simple, and effective treatment for patients with TMD.	Desensitizing TRPV1 receptors and activating central pain-inhibitory pathways.	Low sample size.
4	Komasawa et al. [25]	Jidabokuippo	48 patients/single blind RCT	Powder/VAS	Jidabokuippo (JDI) (7.5 g) vs. control group. No treatment.	Postoperative pain after tooth extraction with mandible bone removal.	Authors reported that JDI administration before general anesthesia effectively decreased the severity of postoperative pain after anesthesia recovery in patients who underwent tooth extraction with mandible bone removal.	Anti-inflammatory, antioxidant, and circulation-enhancing properties.	Small sample. Single center.
5	Maulina et al. [26]	Curcumin	90 patients/RCT	Tablets/numeric rating scale (NRS)	Curcumin vs. mefenamic acid.	Surgical removal of impacted third molars.	Authors reported that curcumin is effective in treating acute inflammation pain in the postsurgical removal of impacted third molars.	Anti-inflammatory, antioxidant, analgesic.	Not reported.
6	Nitecka-Buchta et al. [27]	Cannabis (CBD)	60 patients/double blind RCT	Topical/VAS	Cannabidiol (CBD) formulation vs. control.	TMJ pain.	Authors reported that there was significant improvement in VAS pain in the CBD group compared to placebo in one study at day 14.	Activating cannabinoid receptors ½ thereby inhibiting proinflammatory cytokines.	Not reported.
7	González-Serrano et al. [28]	Propolis	15 patients/double blind RCT split mouth	Topical application/VAS	2% of propolis gel vs. 0.2% of ascorbic acid and 0.2% of tocopherol acetate.	Impacted lower third molar extractions.	Researchers stated that this gel may be effective in preventing alveolitis and thus reducing postoperative pain after impacted third molar extractions.	Antibacterial, anti-inflammatory, antioxidant, healing.	Limited sample. Follow-up compliance. Antibiotic interference.
8	Walczyńska-Dragon et al. [29]	Cannabis (CBD)	60 patients/double blind RCT	Oral administration/VAS	CBD 10% vs. 5% vs. placebo.	Temporomandibular disorders (TMDs)	Authors reported that intraoral use of CBD, specifically 10% CBD has demonstrated superior results compared to 5% CBD.	Acting on cannabinoid CB1/CB2 receptors.	Relatively limited diversity within the study group.
Endodontics									
9	Shabbir et al. [30]	Propolis	52 patients/double blind RCT	Intracanal medicament/VAS	Propolis paste vs. calcium hydroxide.	Postoperative endodontic pain.	Authors suggest that either of these medications can be used as an interappointment medication for the prevention of postoperative pain in a necrotic tooth.	Suppressing lipopolysaccharide (LPS) induced inflammation and inhibiting microbial activity.	Absence of spontaneous pain. No control group.
10	De Grado Gabriel et al. [31]	Eugenol	100 patients/RCT	Topical application/numeric rating scale for pain (NRS)	Drop of articaine from a 40 mg (mL) anesthetic vs. drop of pure eugenol.	Pulpotomy.	Authors reported that choosing pulp dressing, eugenol should be the first choice.	Blocks sodium channels, inhibits prostaglandins, antimicrobial, and anti-inflammatory.	Lack of placebo control.
11	Shen and Yan [32]	Eugenol	92 patients/RCT	Intracanal medication/VAS	Traditional RCT vs RCT combined with eugenol cement.	Acute pulpitis.	Researchers reported that RCT + eugenol cement enhances masticatory function and alleviates toothache.	Suppressing key inflammatory mediators (IL-6, IL-8, TNF-α, leukotriene B4)	Single-center nature of this study. Small sample size.
12	Chrepa et al. [33]	Cannabis (CBD)	61 patients/RCT	Oral administration/VAS	CBD10 (CBD 10 mg/kg), CBD20 (CBD 20 mg/kg), and placebo.	Emergency acute toothache.	Authors reported that oral CBD can be an effective and safe analgesic for toothache.	Activating serotonin (5-HT1A) receptors and modulating inflammatory pathways	Small sample size.
Periodontics									
13	Anil et al. [34]	Curcumin	15 patients/split-mouth study	Mucoadhesive film/numerical rating scale (NRS)	Curcumin mucoadhesive film vs. control placebo mucoadhesive film.	Periodontal postsurgical pain control.	Authors reported that curcumin mucoadhesive film showed promising results in reducing postoperative pain and swelling over 1 week.	Inhibiting COX enzymes, antagonizing TRPV1 channels, and activating potassium ATP channels to modulate pain signaling.	Not reported.
14	Meghana et al. [35]	Turmeric (curcumin)	20 patients/crossover split-mouth randomized study	Periodontal dressing/MVAS, modified visual analog scale	Curcumin gel (Curenext) and noneugenol periodontal dressing (Coe pak).	The secondary outcome was pain assessment postperiodontal surgery.	Researchers reported that periodontal dressing and curcumin are effective in reducing the pain perception.	Lowering inflammation through inhibition of prostaglandins, cytokines, and inflammatory pathways.	Small sample size.
15	Menon et al. [36]	Ginger extract	10 patients/RCT	Oral administration/VAS	Ibuprofen (400 mg) or ginger powder capsules (400 mg) thrice daily for 3 days.	Pain and gingival inflammation following open flap debridement.	Researchers reported that dried ginger powder is as effective as Ibuprofen in controlling pain and gingival inflammation that arises after open flap debridement.	Inhibiting COX and LOX enzymes, lowering inflammatory mediators.	Small sample size, single blind study.
16	Al-Askar et al. [37]	*Curcuma longa*	66 patients/RCT	Tablets/numerical rating scale (NRS) and verbal rating scale (VRS)	Curcumin capsules (200 mg) vs. MA, mefenamic acid (500 mg).	After surgical periodontal therapy.	Researchers reported that compared with MA, curcumin is ineffective in the reduction of pain and discomfort after SPT.	Reduces inflammation by downregulating COX-2, lipoxygenase, and proinflammatory cytokines (IL-1, IL-6, IL-12, TNF-α).	Not reported.
17	Alshibani et al. [38]	*Zingiber officinale*	44 patients/RCT	Tablets/numeric rating scale (NRS)	Ginger (400 mg) and nonsteroidal anti-inflammatory drugs (400 mg).	Nonsurgical Periodontal Therapy	Authors reported that ginger and traditional NSAIDs are effective in reducing postoperative pain and inflammation following NSPT in patients with moderate periodontitis.	Reducing inflammatory cytokines IL-6, IL-1β, and TNF-α.	All participants had moderate periodontitis.
18	Oliveira et al. [39]	Green tea	42 patients/RCT	Gel/VAS	Gel containing green tea extract vs. hyaluronic acid (HA)	Palatal wounds after FGG removal	Researchers reported that gel containing green tea extract and HA application in palatal wounds after FGG removal does not provide clinical healing benefits.	Anti-inflammatory and antioxidant properties.	Small sample size. Short evaluation period.
Oral medicine									
19	Tavangar et al. [40]	*Punica granatum*	60 patients/double blind RCT	Gel/paste/VAS	*Punica granatum* formulated gel vs. triadent oral paste vs. placebo.	RAS	Researchers reported that *Punica granatum* gel has a successful effect in controlling and treating recurrent aphthous stomatitis.	Antioxidant, anti-inflammatory, antibacterial, and wound-healing properties.	Small sample size.
20	Bakhshi et al. [41]	Curcumin gel	48 patients/double blind RCT	Gel/VAS	1% curcumin nanomicelle gel or 2% curcumin gel,	RAS	The authors stated that 1% curcumin nano gel can be effectively used to enhance the healing of RAS.	Inhibition of inflammation and oxidative stress.	Small sample size.
21	Lavaee et al. [42]	*Punica granatum*	30 patients/crossover RCT	Tablets/paste/VAS	Pomegranate (PG) flower tablets vs. triadent oral paste.	Minor RAS	Authors reported that the PG flower tablet is more effective in RAS.	Reduces cytokines, oxidative stress, and microbes.	Limited sample size. No long-term patient follow-up.

**Table 2:** Summary of findings table (GRADE approach).

S. no.	Outcome/phytotherapeutic agent	Number of studies (design)	Effect on pain reduction	Certainty of evidence (GRADE)	Key considerations
1	Eugenol (clove oil)	3 RCTs	Consistently reduced pain in alveolar osteitis, pulpotomy, and pulpitis compared to placebo or standard care.	**Moderate** ⬤⬤⬤◯	Downgraded for some concerns in risk of bias; upgraded for consistency of findings.
2	Curcumin (turmeric)	5 RCTs	Mixed results: effective in some formulations (nanomicelle, mucoadhesive films), but not consistently superior to NSAIDs.	**Low** ⬤⬤◯◯	Downgraded for inconsistency and inaccuracy (small samples, variable formulations).
3	Ginger (*Zingiber officinale*)	3 RCTs	Comparable analgesic effect to ibuprofen in postsurgical and periodontal pain.	**Moderate** ⬤⬤⬤◯	Consistent across studies, but limited by small sample sizes.
4	Cannabidiol (CBD)	3 RCTs	Significant pain reduction in TMD and emergency toothache, dose-dependent effect.	**Low to moderate** ⬤⬤◯◯ to ⬤⬤⬤◯	Downgraded for small sample sizes and short follow-up; promising but emerging evidence.
5	Propolis	2 RCTs	Reduced postextraction and endodontic pain, comparable to calcium hydroxide.	**Low** ⬤⬤◯◯	Downgraded due to limited sample size and pilot study design.
6	*Punica granatum* (pomegranate)	2 RCTs	Reduced pain in recurrent aphthous stomatitis.	**Low** ⬤⬤◯◯	Downgraded for inaccuracy, small samples, and short-term outcomes.
7	Green tea extract	1 quasi-RCT	Did not significantly improve pain after free gingival graft.	**Very low** ⬤◯◯◯	Downgraded for risk of bias, inaccuracy, and inconsistency.

## Data Availability

Data sharing is not applicable to this article as no datasets were generated or analyzed during the current study.

## Supporting Information

Supporting Information 1 Search strategy used for the literature review across Google Scholar, PubMed, and Scopus databases, including keywords and Boolean operators.

Supporting Information 2 Screenshot of the results retrieved from Scopus, PubMed, and Google Scholar databases using Publish or Perish software.
