# Supplementary material for: Analgesic Efficacy of Phytotherapeutic Agents in Dental Pain Management: A Systematic Review
Source: Int J Dent. 2025 Nov 20;2025:5614623. doi: 10.1155/ijod/5614623 (PMC12659986; doi:10.1155/ijod/5614623)
Supplement: Supporting Information 1 — Search strategy used for the literature review across Google Scholar, PubMed, and Scopus databases, including keywords and Boolean operators. [file 5614623.f1.docx]

**Search strategy**

| Google scholar | Plant-based extracts”\|phytotherapy\|“herbal therapy”\|“herbal medicine”\|“plant-derived natural extracts”\|“medicinal plants”\|“Pain relief”\|analgesia\|“pain alleviation”\| “Dental pain”\| “Oral pain”\|“Endodontic pain”\|Periodontal pain”\|“ extraction pain”\|“oral ulcer pain” |
| --- | --- |
| Pubmed | Plant-based extracts*” OR “phytotherapy” OR “herbal therapy* OR “herbal medicine*” OR “plant-derived natural extracts” OR “Pain relief*” or “analgesia” OR “pain alleviation*” OR “Dental pain*”OR “Oral pain*” OR “Endodontic pain*” OR “Periodontal pain*” OR “extraction pain*” OR “oral ulcer pain*” |
| Scopus | Plant-based extracts OR phytotherapy OR herbal therapy OR herbal medicine or plant-derived natural extracts OR AND Pain relief OR analgesia OR pain alleviation AND Dental pain OR Oral pain OR Endodontic pain OR Periodontal pain OR extraction pain OR oral ulcer pain |
