# Supplementary material for: Analgesic Efficacy of Phytotherapeutic Agents in Dental Pain Management: A Systematic Review
Source: Int J Dent. 2025 Nov 20;2025:5614623. doi: 10.1155/ijod/5614623 (PMC12659986; doi:10.1155/ijod/5614623)
Supplement: Supporting Information 2 — Screenshot of the results retrieved from Scopus, PubMed, and Google Scholar databases using Publish or Perish software. [file 5614623.f2.docx]

**Supplementary File 2**

**Screenshot of results in Scopus, PubMed and Google Scholar databases using Publish and Perish software.**


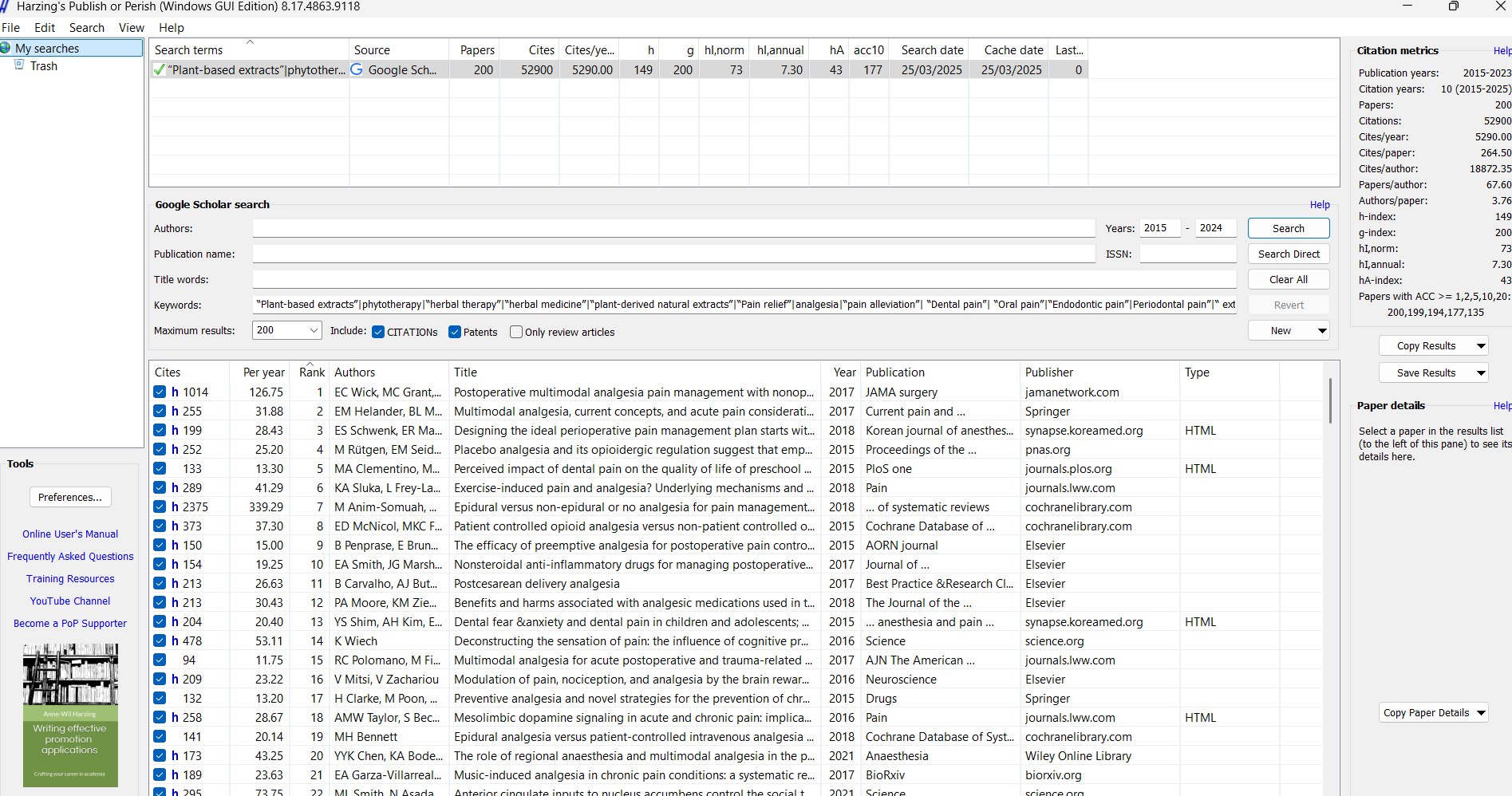


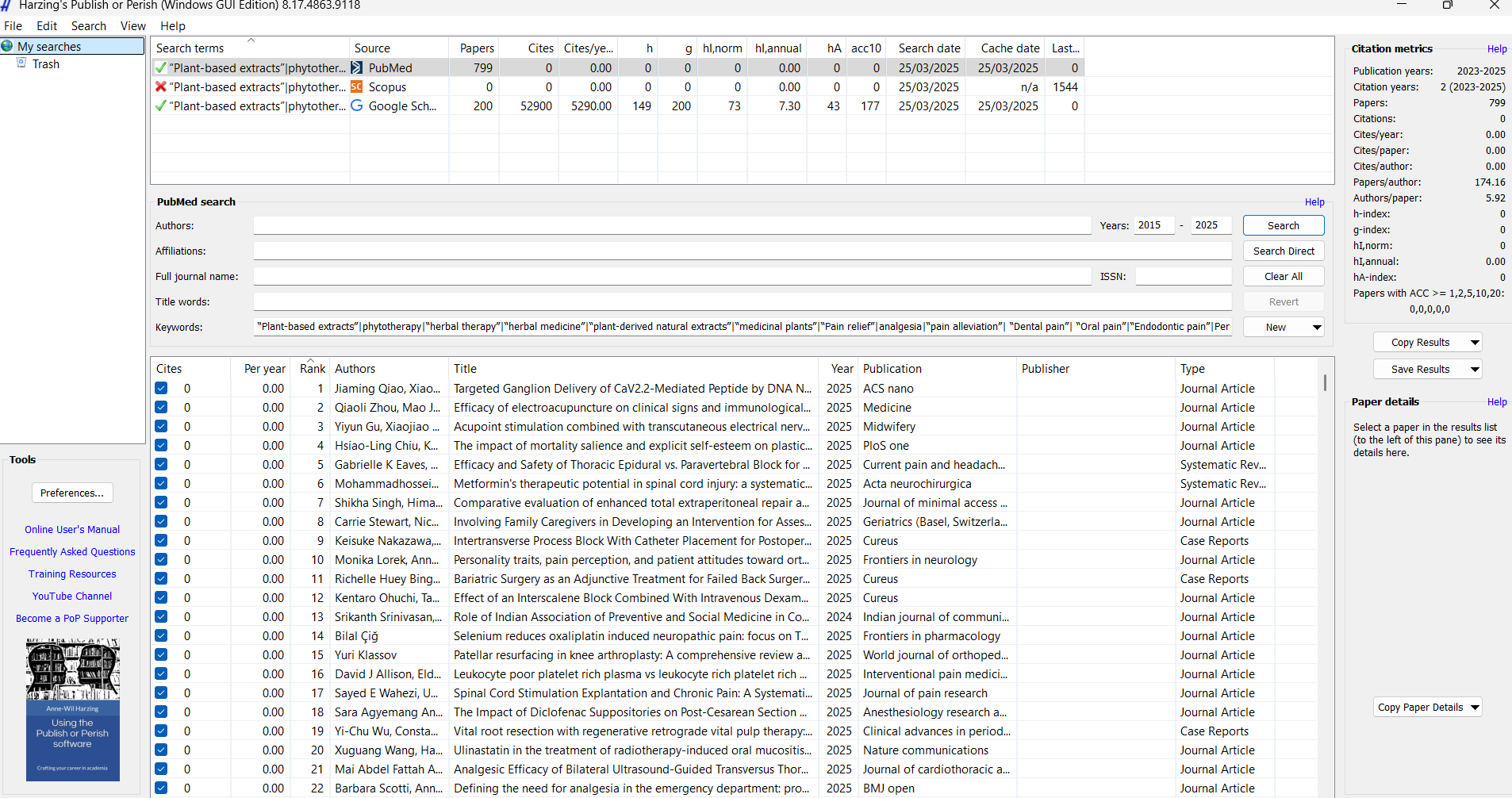


Articles were retrieved from Scopus, PubMed and Google Scholar databases

Reference:

Harzing, A.W. (2007) Publish or Perish, available from <https://harzing.com/resources/publish-or-perish>.
